# The development of acquired equivalence from childhood to adulthood—A cross-sectional study of 265 subjects

**DOI:** 10.1371/journal.pone.0179525

**Published:** 2017-06-20

**Authors:** Gábor Braunitzer, Attila Őze, Gabriella Eördegh, Anna Pihokker, Petra Rózsa, László Kasik, Szabolcs Kéri, Attila Nagy

**Affiliations:** 1 Nyírő Gyula Hospital, Laboratory for Perception & Cognition and Clinical Neuroscience, Budapest, Hungary; 2 University of Szeged, Faculty of Medicine, Department of Physiology, Szeged, Hungary; 3 University of Szeged, Faculty of Dentistry, Department of Operative and Esthetic Dentistry, Szeged, Hungary; 4 University of Szeged, Faculty of Arts, Institute of Education, Department of Social and Affective Education, Szeged, Hungary; Waseda University, JAPAN

## Abstract

Acquired equivalence (AE) is a form of feedback-based associative learning where the subject learns that two or more stimuli are equivalent in terms of being mapped onto the same outcomes or responses. While several studies dealt with how various neurological and psychiatric conditions affect performance on AE tasks (typically with small populations), studies dealing with AE in healthy subjects are rare, and no study has ever made an attempt to plot the development of this form of learning from the childhood through adulthood. In a cross-sectional study, we assessed the AE performance of 265 healthy subjects aged 3 to 52 years with the computer-based Rutgers Equivalence Test (Fish-Face Test, FFT). The test assesses three main aspects of AE: the efficiency of pair learning, the efficiency of the retrieval of acquired pairs, and the ability to generalise previous knowledge to a new stimulus that partially overlaps with the previous ones. It has been demonstrated in imaging studies that the initial, pair learning phase of this specific test is dependent on the basal ganglia, while its generalization phase requires the hippocampi. We found that both pair learning and retrieval exhibited development well into adulthood, but generalisation did not, after having reached its adult-like level by the age of 6. We propose that these findings might be explained by the integrative encoding theory that focuses on the parallel dopaminergic midbrain-striatum/midbrain-hippocampus connections.

## Introduction

Acquired equivalence (AE) is a form of learning where generalisation is increased between two superficially dissimilar stimuli that have previously been associated with similar outcomes. In other words, the learning organism learns that two or more stimuli are equivalent in terms of being mapped onto the same outcomes or responses [1]. An elementary form of cognitive processing as it may well be, AE has been associated with complex cognitive phenomena, such as the guilt/honor by association fallacy, that is, when a negative or positive quality of a person or object transfers to another person or object, merely by co-occurrence [2]. AE has traditionally been studied in pigeons [3–7], but the literature of the last one and a half decades has demonstrated that this kind of cognitive processing is also characteristic of humans [1, 2, 8, 9], and it shows characteristic deficits in various diseases and conditions that affect the central nervous system [8, 10–13]. Probably the most direct demonstration of this was delivered by Myers and colleagues, who compared the performance of patients with Parkinson's disease and hippocampal atrophy in a computerised AE paradigm [8]. Basically, the paradigm consisted of a pair association and a generalisation phase, and the authors found that performance on the former was deficient in Parkinson's disease, and the latter in hippocampal atrophy. The observation allows the conclusion that the striatum and the hippocampi are structures of key importance for association and generalisation, respectively. The role of the hippocampi in generalisation is also corroborated by imaging studies of healthy subjects [14]. It is remarkable that while the disease-related deficits have been described by comparison to healthy control subjects, we know relatively little about the development of AE in healthy subjects, as the available studies are only a few and difficult to compare. The latest study on this topic is by Simon and Gluck [15], who compared college-aged and older healthy adults, and found an age-related decline of performance, but they did not cover younger ages. Earlier studies [9, 16] concentrated on children, but they are difficult to compare because of methodological differences. In some cases, the low number of participants is also a problem [9]. Studying AE across the lifespan, though, is important because of age-dependent structural and functional changes to the striatum and the hippocampi [17–19]. In addition, in the case of the hippocampi, this development is often claimed to be influenced by sex [20], but this is a matter of debate [21]. If such an effect does exist, it may be reflected in the development of generalization. No previous studies found such an effect, but this can be due to the low number of participants.

The AE paradigm developed by Myers and co-workers (also known as the fish-face paradigm, FFP) [8] is an entirely computer-based one, which is easy to use, easy to interpret, and given that it can be administered anytime and anywhere on a laptop, it is optimal for the assessment of larger numbers of subjects. Furthermore, its playful format (the task is to pair cartoon faces with colored fish) makes it an optimal tool for the assessment of children from the youngest ages. Our research group has worked with this paradigm since 2006, mostly with the purpose of the assessment of AE in various conditions, from Alzheimer's disease to migraine [12, 22–24]. Having realised, though, that we did not have normative data about the age-dependence and the sex-dependence of AE, we started to gather data for a developmental study from healthy subjects in 2014. Data were gathered from altogether 265 healthy subjects aged between 3 and 52 years. In this study we present our findings and observations regarding the development of AE, as assessed by FFP.

The specific aim of the study was to determine how performance in the various phases of FFP changes with age, and thereby providing the first developmental description of acquired equivalence and its components [i.e. association and generalisation]. Imaging evidence unequivocally suggests that both the basal ganglia and the hippocampi develop well into adulthood, and that especially the hippocampi show a dynamic remodeling throughout the human life [17–19]. Therefore, we hypothesised that both association and generalisation performance would exhibit development in childhood and adolescence, to reach an optimum in adulthood. As for the sex aspect, we expected to find developmental differences between males and females, especially in generalisation, given the widely accepted view that hippocampal development is sex-dependent [20]. Furthermore, we hypothesised that generalisation performance would depend on association learning performance.

## Materials and methods

### Participants

Altogether 265 healthy subjects were assessed (n_female_ = 149, n_male_ = 116, age range: 3–52 years). The subjects were recruited on a voluntary basis. All subjects were of the Caucasian race and of similar socioeconomic status (middle class). The subjects were recruited from a kindergarten, an elementary school, two high schools and from among the academic staff of the University of Szeged, on a voluntary basis. The final sample size and the size of the subsamples were determined by the number of volunteering subjects in the 2 years of data collection. Only persons free of any ophthalmological, neurological and psychiatric conditions were eligible. The potential subjects (in the case of minors also their parents) were informed about the background and goals of the study, as well as about the procedures involved. It was also emphasised that given the lack of compensation or any direct benefit, the participants were free to quit at any time without any consequence (no one did so). Those who decided to volunteer signed an informed consent form. When minors were assessed, their parents signed the informed consent form, as required by the Hungarian law.

The study protocol conformed to the tenets of the Declaration of Helsinki in all respects, and it was approved on several occasions by the Regional Research Ethics Committee for Medical Research at the University of Szeged, Hungary.

### Methods and study protocol

The tests were run on a Lenovo ThinkPad T430 laptop computer and two iBook G3 "Clamshell" laptop computers. The testing software (originally written for iOS) was used and rewritten in Assembly (for Windows) with the written permission of Myers and colleagues at Rutgers University, NJ. The testing sessions took place in a quiet room with the subjects sitting at a comfortable distance from the computer screen. One subject was tested at a time, and no time limit was set so that the subjects could concentrate on the task.

**Fig 1:**
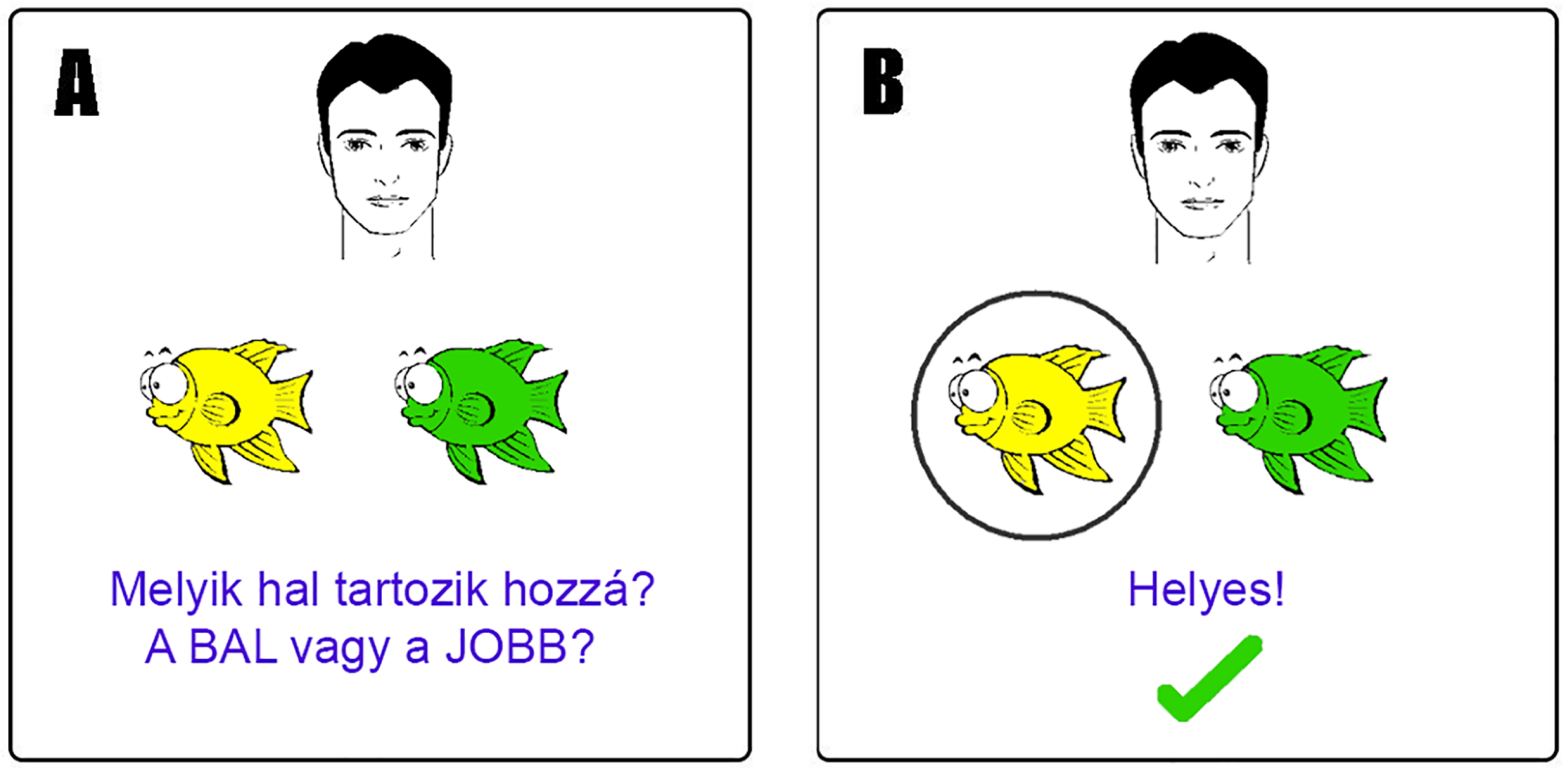
Example screen events during one trial. (A) Stimuli appear. (B) Participant responds and corrective feedback is given. The Hungarian instructions: (A) Which fish belongs to him? The LEFT or the RIGHT one? (B) Correct! The fish in the actual test are colored, the figure is only for illustration purposes.

The testing was done according to Myers et al. [6]. On each trial of the task, participants saw a cartoon face and a pair of fish, and had to learn through trial and error which of the fish went with which face (Fig 1).

There were four cartoon faces (A1, A2, B1, B2) and four possible fish of different colours (X1, X2, Y1, Y2), referred to in the terminology of Myers and colleagues (2003) as antecedents and consequents, respectively. The four possible faces were: a male adult, a male child, a female adult and a female child. The four colours were: red, green, blue and yellow. The antecedent-consequent pairings were randomly generated by the computer from these stimuli for each participant.

To illustrate the process in simple terms: let us assume that the male child (A1) and the female adult (A2) are first associated with the green fish (X1), while the female child (B1) and the male adult (B2) are associated with the red fish (Y1). These are the phases of shaping and equivalence training. This way, the male child and the female adult become associated through the green fish (A1, A2 → X1), and the female child and the male adult through the red fish (B1, B2 → Y1). In the next step, the participant learns that the male child (A1) and the female child (B1) are also associated with the yellow fish (X2) and blue fish (Y2) respectively. This is the phase when the new consequents are introduced. If the equivalence of stimuli has been successfully learned, the participant should be able to generalise that the female adult (A2) is associated with the yellow fish (X2) and the male adult (B2) is associated with the blue fish (Y2). This is what the equivalence testing phase seeks to test. In fact, the situation is analogous to drawing the conclusion of a three-premise syllogism. A formal summary of the process is given in Table 1.

**Table 1:** Summary of the applied paradigm (after Myers et al., 2003). The minimum number of trials to pass each association phase was 41. The number of trials in the generalisation phase was set (48 trials).

*Association Phase 1*: *Shaping*	*Association Phase 2*: *Equivalence Training*	*Association Phase 3*: *New Consequents*	*Generalisation Phase*: *Equivalence testing*
A1 → X1		A1 → X1	A2 → X2?
	A1 → X1	A2 → X1	
	A2 → X1	A1 → X2	
B1 → Y1		B1 → Y1	B2 → Y2?
	B1 → Y1	B2 → Y1	
	B2 → Y1	B1 → Y2	
	**ACQUISITION**		**TESTING**

While the formal description may make the impression that the task is a difficult one, in fact, healthy children [9] and also mentally retarded individuals [25, 26] reliably make this kind of generalisation.

The participants' task throughout the association and generalisation phases was to indicate their choice in each trial by pressing one of two keyboard buttons labeled LEFT and RIGHT. The correct key was uncorrelated with the fish, that is, participants learned that a given face was associated with a fish of a given colour, and not a given key. Visual feedback on the correctness of choice was provided in the acquisition phases but not in the testing phase. New associations were introduced one by one during the acquisition part of the test. New associations were presented mixed with trials of previously learned associations. The subjects had to achieve a certain number of consecutive correct answers after the presentation of each new association to be allowed to proceed. This number was 4 when the first association was presented, and it was increased by 2 upon the presentation of each association that followed- up to 12. From this follows that the length of the association phases varied among the participants, depending upon how efficiently they learned. The generalisation phase, in contrast, always contained 48 trials (12 trials of new and 36 trials of previously learned associations).

### Statistical analysis

Statistical analysis was performed in SPSS 21.0 (IBM, USA), except for the power calculations, which were done in G*Power 3.1.9.2. (Universität Düsseldorf, Germany). The results were analyzed in three groups: results from the association phases, results from the "old associations" part of the generalisation phase (i.e. when the participant was presented an already learned association), and results from the generalisation trials (i.e. previously not learned associations). The number of correct and wrong answers were recorded in all phases, as well as the ratio of these numbers to the total number of trials during a given phase. The number of trials necessary for the completion of the association phases was also recorded. The results were analysed with factorial ANOVA in fourteen cohorts. Sex and cohort were selected as predictors. Cohort 0 involved kindergarten children (3 to 6 years of age), cohorts 1 to 8 corresponded to the grades of the elementary school (7 to 14 years of age), cohort 9 involved high school students (15 to 19 years of age), and cohorts 10–13 involved adults aged 20 to 29, 30 to 39, 40 to 49, and 50+, respectively. The kindergarten cohort was not divided into further subgroups because of the small number of subjects (n = 12), and the high school cohort was dominated by seventeen-year-olds to such an extent that it would have made no sense to create subgroups. Achieved power was calculated in G*Power (Universität Düsseldorf, Germany).

## Results

The achieved power for the factorial ANOVA was 0.88 (f = 0.25, α = 0.004, sample size = 265, number of groups = 14). Cohort-wise performance means by the studied parameters are given in Table 2.

**Table 2:** Cohort-wise performance by the studied parameters.

Cohort	Mean age	N	NAT	ALER	RER	GER
0	4.17 yrs	12	122.33(63.66)	0.31(0.15)	0.29(0.16)	0.40(0.20)
1	7.15 yrs	18	56.78(11.21)	0.10(0.05)	0.10(0.16)	0.18(0.23)
2	8.43 yrs	14	69.00 (42.28)	0.09(0.08)	0.05(0.10)	0.14(0.28)
3	9.60 yrs	22	71.91 (35.49)	0.15(0.20)	0.05(0.09)	0.14(0.30)
4	10.50 yrs	12	59.75(18.30)	0.08(0.06)	0.06(0.09)	0.19(0.26)
5	11.53 yrs	18	71.28(62.61)	0.09(0.14)	0.09(0.15)	0.20(0.28)
6	12.52 yrs	17	85.59(54.27)	0.12(0.11)	0.09(0.09)	0.24(0.28)
7	13.59 yrs	17	69.88(57.68)	0.10(0.12)	0.11(0.19)	0.27(0.32)
8	14.62 yrs	21	89.24(75.09)	0.12(0.12)	0.11(0.15)	0.21(0.28)
9	16.77 yrs	41	63.08(22.73)	0.09(0.09)	0.06(0.07)	0.16(0.26)
10	24.11 yrs	27	59.93(11.69)	0.07(0.04)	0.04(0.08)	0.24(0.34)
11	34.06 yrs	17	53.59(10.24)	0.05(0.03)	0.01(0.01)	0.21(0.31)
12	45.30 yrs	23	59.74(14.77)	0.08(0.05)	0.05(0.05)	0.18(0.28)
13	50.66 yrs	6	79.67 (33.82)	0.13(0.07)	0.07(0.09)	0.03(0.04)

Abbreviations: NAT- number of acquisition trials; ALER- association learning error ratio (the ratio of incorrect choices during the acquisition trials); RER- retrieval error ratio; GER- generalization error ratio. Values are given as mean (SD).

### Number of acquisition trials

The factorial ANOVA analysis of this parameter with cohort and sex as covariates yielded the following results: Sex had no significant effect (F(1,265) = 3.433, p = .07, two-tailed), however, cohort did (F(13,256) = 2.505, p< .001, two-tailed). Their interaction was not significant (F(13,254) = .701, p = .76, two-tailed). A Tukey's post-hoc analysis was conducted on cohort to find out about the source of the significant overall variance. The post-hoc analysis revealed that cohort 0 differed significantly from all other cohorts at different levels of probability (p = .05- .001).

**Fig 2:**
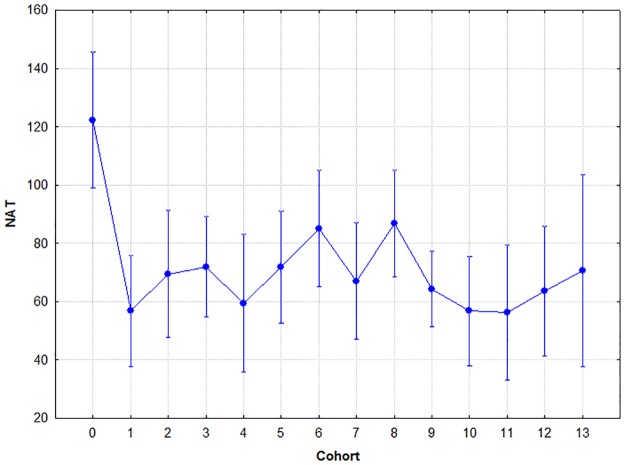
The mean number of trials needed to acquire the associations by cohort. Circle: mean; whiskers: ±SD.

That is, kindergarten children needed significantly more trials to acquire the associations than members of any of the other cohorts. The results are summarised in Fig 2.

### Association learning error ratio

**Fig 3:**
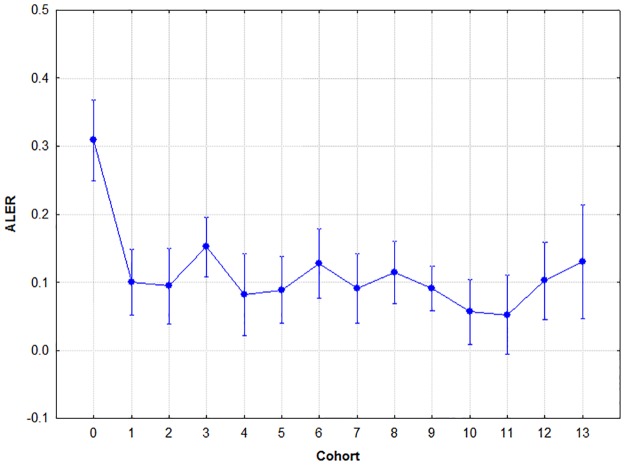
Mean association learning error ratios by cohort. Circle: mean; whiskers: ±SD.

Sex did not have a significant effect on this parameter (F(1,265) = 3.690, p = .06, two-tailed), but cohort did (F(13,256) = 2.505, p< .001, two-tailed). Their interaction was not significant (F(13,254) = 1.253, p = .24, two-tailed). A Tukey's post-hoc analysis was conducted on cohort to find out about the source of the significant overall variance. The post-hoc analysis revealed that cohort 0 differed significantly from all other cohorts at p< .001. In other words, kindergarten children made significantly more mistakes during acquisition than members of any of the other cohorts, and no significant differences were found among the rest of the cohorts. The results are summarised in Fig 3.

### Retrieval

**Fig 4:**
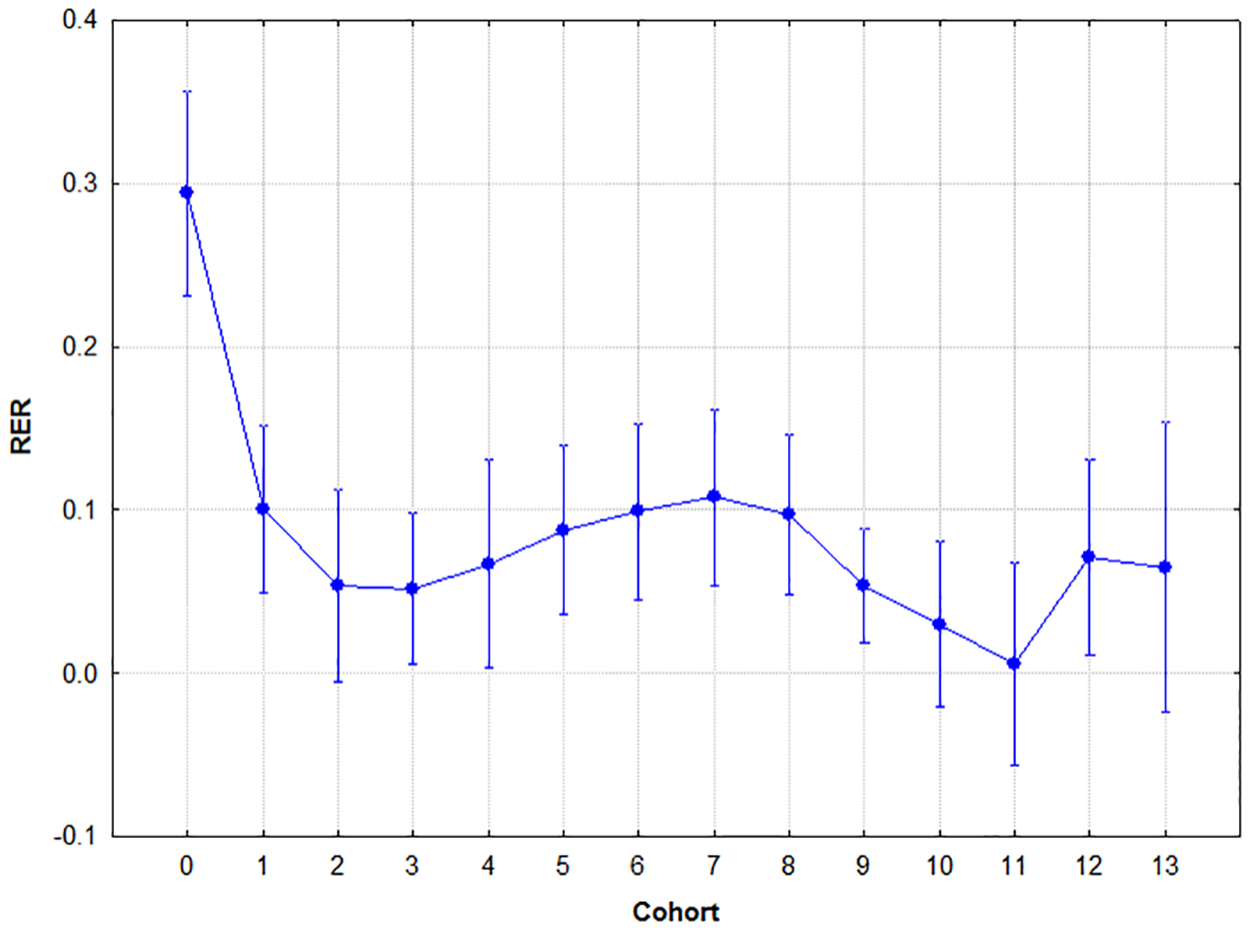
Mean retrieval error ratios by cohort. Circle: mean; whiskers: ±SD.

Sex did not have a significant effect (F(1,265) = 2.950, p = .09, two-tailed), but cohort did (F(13,256) = 4.757, p< .001, two-tailed). Their interaction was not significant (F(13,254) = 1.157, p = .31, two-tailed). A Tukey's post-hoc analysis was conducted to find out about the source of the significant overall variance. The post-hoc analysis revealed that cohort 0 differed significantly from all other cohorts at p< .001. This means that kindergarten children made significantly more mistakes during retrieval than members of any of the other cohorts, and no significant differences were found among the rest of the cohorts. The results are summarised in Fig 4.

### Generalisation

**Fig 5:**
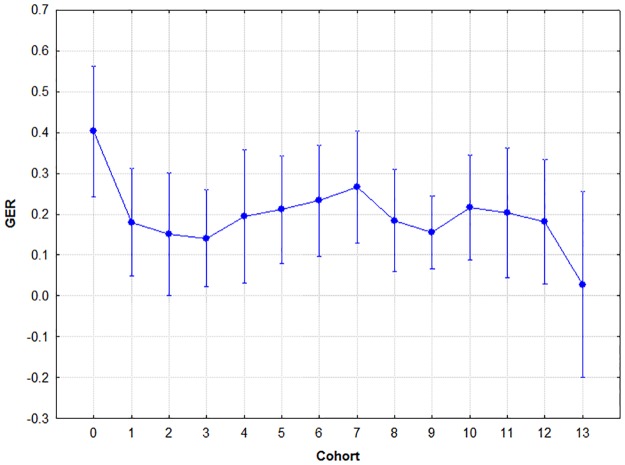
Mean generalisation error ratios by cohort. Circle: mean; whiskers: ±SD.

Factorial ANOVA indicated no significant effect of either sex (F(1,265) = .099, p = .75, two-tailed) or cohort (F(13,265) = .934, p = .52, two-tailed). Neither was their interaction significant (F(13,265) = .601, p = .85, two-tailed). Thus no post-hoc analysis was conducted. The success of generalisation was fairly constant in the studied period. The results are summarised in Fig 5.

### Additional analyses

We also wanted to know if the efficiency of acquisition (NAT, ALER) or the efficiency of retrieval (RER) had a significant effect on the success of generalisation (GER). A multiple regression analysis was performed with GER as the dependent variable and NAT, ALER and RER as the independent variables. Neither NAT (β = -.004, p = .965) nor ALER (β = .021, p = .829) proved to be significant predictors of GER, while RER was a highly significant predictor (β = .503, p< .001). ALER also had a significant effect on RER (β = .673, p < .001), suggesting that the less mistakes a subject made during acquisition, the more likely it was that they would successfully retrieve the stimulus pairs during testing—and the more efficient retrieval was, the more likely it became that the subject would generalise successfully.

**Fig 6:**
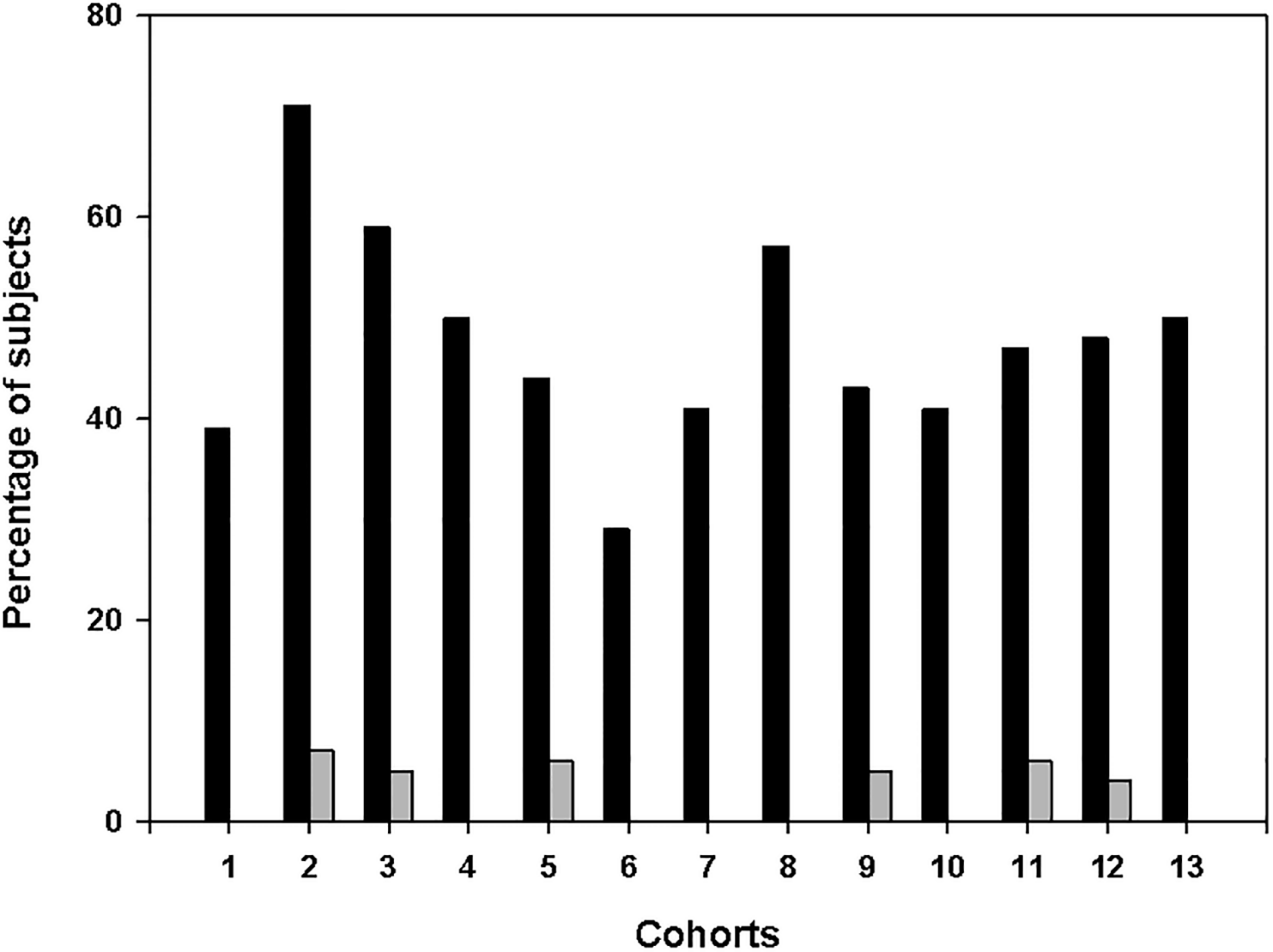
Generalisation performance by cohort. **Performance** expressed as the ratio of 100% correct and 100% incorrect responders. Black- 100% correct, grey- 100% incorrect.

A further way to characterise the efficiency of equivalence acquisition is to calculate the percentage of subjects in each cohort who failed to give correct responses altogether (no generalisation or erroneous rule abstraction) and who made no mistakes at all (stable generalisation) in the test phase. Fig 6 shows these percentages by cohort. The high ratio of 100% correct responses in each cohort except for the youngest one is notable (mean: 44.21%). In contrast, 100% incorrect responses appeared only in a few cohorts, and at percentages below 10% (mean: 2.36%). A chi square analysis (100% correct responders vs. cohort) also supported the cohort-independence of generalisation performance (χ^2^ = 20.38, df = 13, p = 0.1).

Finally, considering the high ratio of subjects who reached ceiling, we wished to make sure that it was not the ceiling effect that was reflected in the overall results. Additional ANOVA analyses were performed without the results of those who reached ceiling (for ALER, RER and GER). These analyses confirmed the original results: only cohort had a significant effect, and only in the case of ALER and RER (ALER p< .001; RER p< .05; GER p = .951)

## Discussion

### The effect of age

As for the effect of age, we hypothesised that both association and generalisation performance would exhibit development in childhood and adolescence, to reach an optimum in adulthood.

The results show that age had a significant effect on pair acquisition and retrieval in the examined period, but not on generalisation. Only one notable (yet not significant) leap of enhancement was observed (between cohorts 0 and 1), but over six years of age, the average generalisation error ratio stabilised in a narrow range (0.14–0.27), and the cohorts did not differ from each other significantly (see Table 2). That is, our hypothesis regarding generalisation was wrong- age did not have a significant effect on it over the age of six.

Similarly to what was found by Shohamy and Wagner [14], the interindividual variability was rather high, but this did not influence the overall performance to a significant extent. A conspicuous finding was the high ratio of 100% correct responders in each cohort (with the exception of kindergarten children), indicating either highly efficient generalisation over the age of six, or that the task was too easy. Indeed, FFP was originally designed for testing populations with cognitive deficit, but having that in mind, the ratio of participants who reached ceiling in the present study is not extremely high. Also, the more difficult a learning task is, the more likely that one starts to measure additional cognitive factors, like attention, working memory and so on [27], which can have a confounding effect. It is difficult to tell precisely why a high percentage of participants reached ceiling in this study, but the additional factorial ANOVAs (performed without those who reached ceiling) showed that the ceiling effect did not interfere with the results to a significant extent.

### The effect of NAT, ALER and RER on GER

The number of trials necessary for acquisition and the ratio of incorrect responses during acquisition had no significant effect on the efficiency of generalisation. Retrieval error ratio (itself significantly influenced by acquisition error ratio), though, turned out to have a significant effect in this respect. tThis suggests that the success of generalisation is related the most closely to the retrieval of the previously acquired associations, but the retrieval does not have to be maximal for generalisation to happen. This way, 7-year-olds already generalise as well as adults, even if they need more trials to acquire the associations and they have a higher retrieval error ratio. Furthermore, once there is sufficient retrieval to allow generalisation, generalisation will be highly efficient.

### The effect of sex

Sex did not influence the performance in any part of FFP, but it must be noted that the sexes were not completely equated across the cohorts (in cohorts 10, 11 and 12 there was an approximately 75% female dominance). Apart from that problem, it seems that sex does not influence age-dependent performance in FFP, even if the hippocampi, whose development is often described as sex-dependent [20], are crucial to this task. In this respect, our study corroborates the results of earlier studies with smaller samples. It is beyond the limitations of this study to explain why the sex effect is missing. It is possible that the transfer function is simply left unaffected by the dynamic, age- and sex- related structural changes of the hippocampi, but the finding might also be interpreted as behavioral level evidence to suggest that a correlation between sex and hippocampal volume does not exist at all [18].

### A possible explanation for the observed developmental pattern

While it is difficult to measure directly,it seems logical to assume that both explicit and implicit memory are necessary for FFP; during the acquisition phase, when the individual stimulus pairs are learned, explicit memory could support voluntary rule application through the conscious recollection of previously learned pairs. Later on, when the rule of pairing has been acquired to an optimal level, the implicit element becomes dominant.

While it is the hippocampus that is usually pointed out as the key structure in explicit memory [28, 29], feedback (or reinforcement)-driven learning is widely associated with the ventral tegmental area/substantia nigra (VT/SN)- striatum connection in the literature [30–32]. As one of the functions associated with the striatum is the cognitive control of memory (in cooperation with the prefrontal cortex) [33], and as evidence suggests that striatal activity declines across learning when acquiring individual associations in a reinforcement learning context [34], the hypothesis that the initial pair learning and retrieval in test basically differ in the level of voluntary effort involved might be not far-fetched. However, we did not test for voluntary rule application in this study. This might be regarded as a weakness of this study, but we had reasons not to do so One of those was that voluntariness/ consciousness is a concept that is especially difficult to grasp and measure. To name a practical problem, it would not have been easy to explain what we meant by voluntary decision to our youngest subjects, if possible at all—but even if we had managed to explain it, and this is another important reason, generalisation in AE is really tricky in this respect. As Shohamy and Wagner put it: "The present form of generalisation may be thought of as a type of false memory (…), in that participants have the subjective sense of having already experienced the pairing of two elements that in fact had never been encountered together".[14]. Obviously, it is no use asking if the subject was in fact aware of what was going on.

Several studies investigated the developement of other types of learning involving both explicit and implicit learning. Generally, it seems that the key factor in the development of a given type of learning is the ratio of the implicit and explicit elements. The results of several studies suggest that the explicit system goes through significant development until adulthood, while the implicit system is well- developed already at an early age [35–39]. Minda and collagues [38] used a category learning test to compare explicit and implicit learning in children of different ages and adults. They found that 3-year-olds performed at the same level as adults if the task required the involvement of the implicit memory, however, even 8-year-olds performed far below adults if the task required the involvement of the explicit memory. Studies of sequence learning also found that the efficiency of implicit learning does not differ significantly between different age groups; it is rather the explicit elements that are responsible for the differences in performance [35, 36]. Studies of statistical learning yielded similar results [37, 40]. A decrement of the efficiency of the implicit functions was also described in older ages [41–43]. It should be noted, however, that this trend was only observable after the age of 60, and our oldest subjects were younger, hence the lack of decline among the older participants in this study.

But how can or, at least, could the present results be explained? Given the relative paucity of literature on human AE, it would be difficult to offer anything but a hypothesis based on the literature. We argue that our findings may be explained in the framework of the *integrative encoding* account of AE [14, 29]. This account is especially fit for our purposes, as it focuses on the parallel midbrain—striatum and midbrain-hippocampus connections, which FFP is known to depend on [8]. In brief, these parallel connections are assumed to work simultaneously. While the SN- striatum loop serves the voluntary learning of individual stimulus pairs with the help of feedback, the VT- hippocampus loop conveys information to the hippocampus, where a network of all encountered stimuli is constructed, with their connections and overlaps. Then, in the test phase, this network is retrieved or reactivated, which makes both the retrieval of the acquired associations and generalisation possible. According to this account, the previously unlearned stimulus (as a prediction error) would trigger the retrieval/activation of the association network, and its location within the network would be determined by a feature it shares with the elements in the network (e.g. the color of a fish, the perceived gender of a cartoon face). Naturally, we do not believe that after the acquisition phase retrieval is purely implicit and lacking voluntary effort. What we hypothesise is that the ratio of non-conscious rule application becomes higher.

Developmental studies generally agree that the maturation of explicit memory takes more time than that of implicit memory [44–48]. Our results are in agreement with that. We found a significant age-dependent development of acquisition in the examined period, expressed both in the number of trials required and the ratio if incorrect responses. We argue that this reflects the increasing efficiency of pair learning and voluntary recollection. Studies about the protracted development of the human striatum support this [17, 18]. In contrast, we found no development in generalisation. After the age of six, children generalised as well as adults. This is in agreement with the findings that suggest that implicit memory functions adult-like at quite early ages [49]. This finding also points out that even if the hippocampi are usually mentioned in the context of explicit memory, they have a broader function of encoding and retrieval, regardless of whether these happen with conscious effort [50, 51].

In the framework of the integrative encoding theory, efficiency of hippocampal parallel encoding depends partially on the efficiency of the VT/SN-striatum loop. If we accept this, it comes as no surprise that, as we found, the efficiency of acquisition had a significant effect on retrieval in the test phase. Accordingly, test phase retrieval also showed a significant enhancement in the studied period.

Finally, test phase retrieval had a significant effect on generalisation, which, however, did not show any significant enhancement over the age of six. On the one hand this suggests that this specific hippocampal function is not affected by the lifelong dynamic remodeling of the structure [52–54], and on the other, it shows that generalisation happens even when the efficiency of retrieval is low. This way it becomes possible for young children who have trouble learning individual associations, or voluntarily retrieving them to make generalisations without difficulty.

## Conclusions

From our observations we conclude that generalisation, the core element of any acquired equivalence task (and of several more complex cognitive functions), is adult-like quite early in childhood regardless of sex, and it can be highly efficient even when the learning of stimulus pairs and their retrieval are yet to reach reach their optimal levels. We propose that this observation can be explained by the integrative encoding hypothesis, according to which generalisation is supported by a parallel neural network characterised by faster maturation.
